# Natural polyphenols convert proteins into histone-binding ligands

**DOI:** 10.1016/j.jbc.2022.102529

**Published:** 2022-09-24

**Authors:** Kosuke Yamaguchi, Masanori Itakura, Mona Tsukamoto, Sei-Young Lim, Koji Uchida

**Affiliations:** 1 Graduate School of Agricultural and Life Sciences, The University of Tokyo, Tokyo, Japan; 2 Japan Agency for Medical Research and Development, CREST, Tokyo, Japan

**Keywords:** natural polyphenols, histone, oxidative deamination of lysine, posttranslational modification, protein aggregation, oxidation-reduction (redox), BSA, bovine serum albumin, Bt-AET, N-biotinyl-2-aminoethane-1-thiol, Bt-APA, N-biotinyl-5-aminopentylamine, CTH, calf thymus histone, EGCG, (-)-epigallocatechin-3-O-gallate, ESI, electrospray ionization, HRP, horseradish peroxidase, IDR, intrinsically disordered region, LNL, lysinonorleucine, oxVC, oxidized vitamin C, PBST, PBS and Tween 20

## Abstract

Antioxidants are sensitive to oxidation and are immediately converted into their oxidized forms that can react with proteins. We have recently found that proteins incubated with oxidized vitamin C (dehydroascorbate) gain a new function as a histone-binding ligand. This finding led us to predict that antioxidants, through conversion to their oxidized forms, may generally have similar functions. In the present study, we identified several natural polyphenols as a source of histone ligands and characterized the mechanism for the interaction of protein-bound polyphenols with histone. Through screening of 25 plant-derived polyphenols by assessing their ability to convert bovine serum albumin into histone ligands, we identified seven polyphenols, including (-)-epigallocatechin-3-*O*-gallate (EGCG). Additionally, we found that the histone tail domain, which is a highly charged and conformationally flexible region, is involved in the interaction with the polyphenol-modified proteins. Further mechanistic studies showed the involvement of a complex heterogeneous group of the polyphenol-derived compounds bound to proteins as histone-binding elements. We also determined that the interaction of polyphenol-modified proteins with histones formed aggregates and exerted a protective effect against histone-mediated cytotoxicity toward endothelial cells. These findings demonstrated that histones are one of the major targets of polyphenol-modified proteins and provide important insights into the chemoprotective functions of dietary polyphenols.

Polyphenols, the most diverse group of phytochemicals, have attracted a great deal of attention as potential chemopreventive agents for chronic diseases, such as heart disease, cancer, diabetes, stroke, and arteriosclerosis. Polyphenols are generally considered antioxidants. However, because they are susceptible to oxidation, they are easily converted into their oxidized form. Interestingly, some polyphenols, when oxidized, acquire a new function of reactivity with proteins (Fig. 1). For example, green tea polyphenol (-)-epigallocatechin-3-O-gallate (EGCG) is converted to its oxidized form, which covalently binds to lysine residues to form Schiff base intermediates, followed by the lysine deamination product, α-aminoadipic-5-semialdehyde (1). The existence of a unique 6-membered ring 2-piperidinol product as an equilibrium form of α-aminoadipic-5-semialdehyde has recently been established (2). Piceatannol, a naturally occurring hydroxylation analog of the red wine polyphenol resveratrol, mediates protein polymerization through the formation of a lysine-derived crosslink, dehydrolysinonorleucine (3). Some of the oxidized polyphenols have also been shown to form covalent adducts with proteins *via* Michael addition (4, 5, 6). Intriguingly, polyphenol-modified proteins have been shown to acquire new functions, including oxidation-specific epitope activity against natural antibodies (1, 2, 3). Based on these findings, it has been suggested that many of the beneficial effects of polyphenols may be due in part to oxidative modification of proteins by the polyphenols.

**Figure 1:**
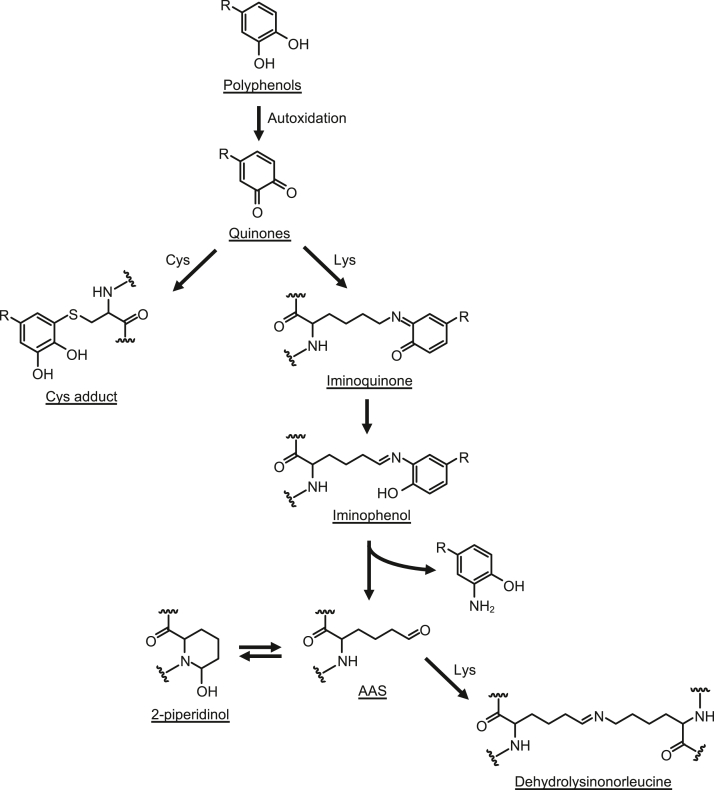
**Protein modification by oxidized polyphenol.** Polyphenols are oxidatively converted to *o*-quinones, which are reactive with nucleophilic amino acid, such as lysine and cysteine. Upon reaction with lysine residues, oxidized polyphenols (*o*-quinones) are covalently attached to lysine residues to form Schiff base intermediates, such as iminoquinone and iminophenol, followed by aminated polyphenols and deaminated products (AAS). AAS is equilibrated with six-membered ring 2-piperidinol. Crosslinks are formed by Schiff base reactions between AAS and the ε-amino groups of unoxidized lysine residues. AAS, α-aminoadipic-5-semialdehyde.

Histones are the major protein component of chromatin and act as spools around which DNA wraps and play a role in gene regulation. They are also expressed on the cell membranes of inflammatory cells, such as macrophages and monocytes, and act as plasminogen receptors to promote the inflammatory response (7, 8). In addition, they are present in the extracellular space released from both damaged and activated cells. These extracellular histones act as components of neutrophil extracellular traps and contribute to biological defense by trapping entangled bacteria (9). On the other hand, the extracellular histones are known to be involved in the pathogenesis of sepsis, acute kidney injury due to rhabdomyolysis, and chronic vascular disease (10, 11, 12). In fact, extracellular histone H4 has been shown to mediate membrane lysis of smooth muscle cells, causing damage and inflammation of arterial tissue (12). Our recent study found that proteins modified with oxidized vitamin C (oxVC) function as histone ligands (13). In addition, oxVC-modified proteins inhibit the binding of plasminogen to the histone component H2B and regulate the recruitment of monocytes/macrophage to the site of inflammation. The discovery of histones as cell surface receptors for the oxVC-modified proteins suggested that, in addition to the general concept of oxVC-modified proteins as danger-associated molecular patterns that mediate proinflammatory responses, they may also be involved in the homeostatic response *via* binding to histones. The findings led to an attractive hypothesis that antioxidants by conversion to the oxidized form may generally have similar functions. This study extends the research into the development of antioxidant functions and establishes that oxidation-sensitive polyphenols can also be a source of histone-binding ligands. Furthermore, it provides details about the mechanism of interaction between polyphenol-modified proteins and histones.

## Results

### Polyphenol-modified proteins acquire histone binding activity

**Figure 2:**
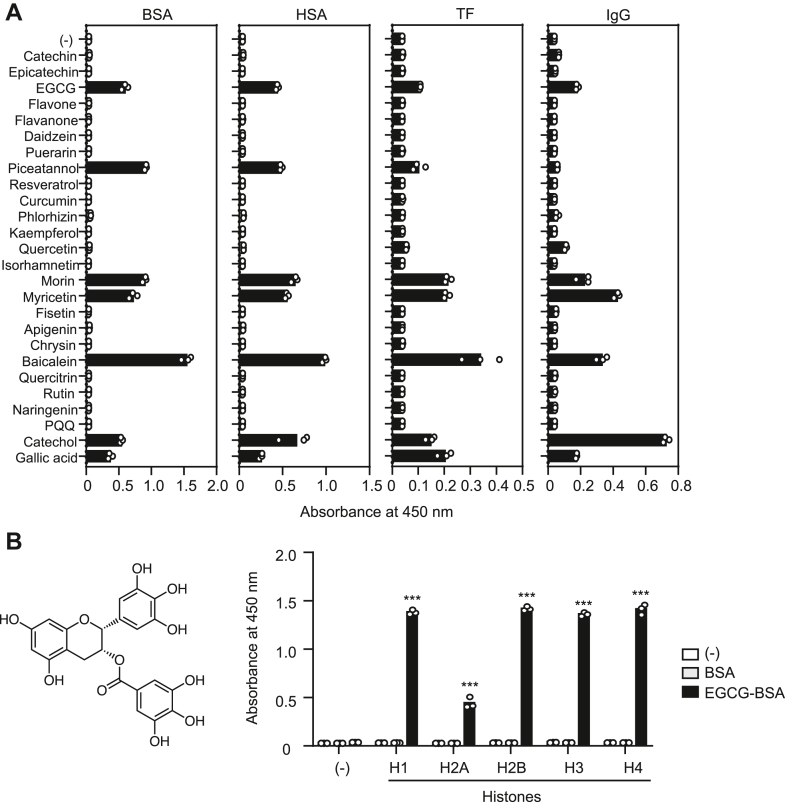
**Polyphenol-modified proteins acquire histone-binding activity.***A*, binding of polyphenol-modified proteins to CTH. CTH immobilized on ELISA plate were incubated with Bt-polyphenol-modified proteins (10 μg/ml). The binding was detected using streptavidin-HRP. *B*, binding of EGCG-BSA to the recombinant histones. Recombinant histones immobilized on ELISA plate were incubated with biotin-labeled BSA (Bt-BSA) or EGCG-modified Bt-BSA (EGCG-Bt-BSA) (10 μg/ml). The binding was detected using streptavidin-HRP. BSA, bovine serum albumin; CTH, calf thymus histone; EGCG, (-)-epigallocatechin-3-O-gallate; HRP, horseradish peroxidase.

To evaluate the binding of polyphenol-modified proteins to histones, we selected albumins (bovine serum albin [BSA] and human serum albumin), transferrin, and immunoglobulin (IgG) as model proteins abundant in blood. They were incubated with various polyphenols (chemical structures in Fig. S1) *in vitro* and evaluated the binding potential to calf thymus histone (CTH) by a solid-phase binding assay. The screening of 25 polyphenols by assessing their ability to convert BSA to CTH ligands identified seven polyphenols, including EGCG, piceatannol, and baicalein (Fig. 2*A*). We then examined the binding specificity of the EGCG-modified proteins toward five major families of histone proteins (H1, H2A, H2B, H3, and H4) *in vitro*. The solid-phase binding assay revealed that the EGCG-modified BSA (EGCG-BSA) was almost equally bound to the histones except for H2A (Fig. 2*B*). These results revealed that some of the polyphenols contained in dietary fruits and vegetables could convert serum proteins into the histone-binding partners.

### N-terminal region of histone H2B contributes to the binding of polyphenol-modified proteins

**Figure 3:**
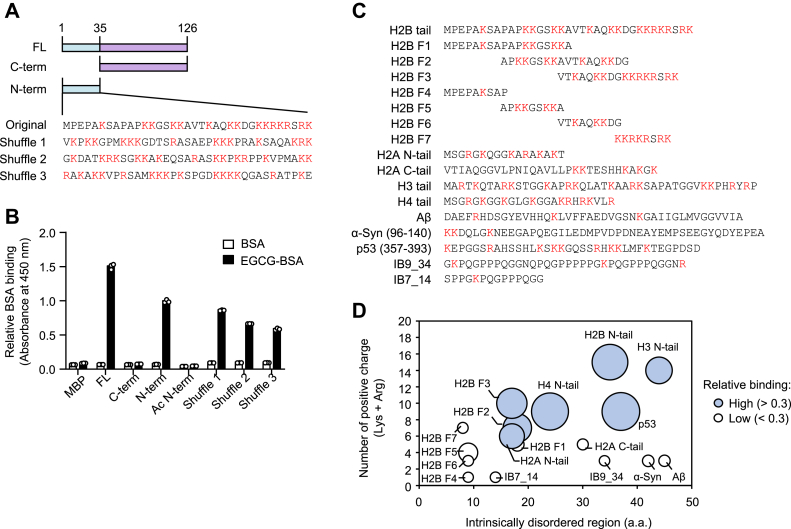
**N-terminal region of histone H2B contributes to EGCG-BSA binding.***A*, schematic representation of full-length, C-terminal globular domain, N-terminal tail domain, and N-terminal tail-shuffled sequences of H2B. *B*, binding of EGCG-BSA to MBP-fused H2B. MBP-fused H2B immobilized on ELISA plate were incubated with Bt-BSA or EGCG-Bt-BSA (10 μg/ml). The binding was detected using streptavidin-HRP. *C*, schematic representation of MBP-fused IDR peptides. *Red* characters indicate positively charged amino acids (lysine and arginine). *D*, binding of EGCG-BSA to MBP-fused IDRs. MBP-fused IDRs immobilized on ELISA plate were incubated with Bt-BSA or EGCG-Bt-BSA (10 μg/ml). The binding was detected using streptavidin-HRP. The binding ability was normalized by EGCG-BSA binding to H2B N-terminal tails and relative bindings are shown. The *blue* bubbles indicate IDRs showing relatively high binding to EGCG-BSA. Binding of EGCG-BSA to positively charged proteins. BSA, bovine serum albumin; EGCG, (-)-epigallocatechin-3-O-gallate; HRP, horseradish peroxidase; IDR, intrinsically disordered region.

The results that the EGCG-modified proteins bound to histone proteins regardless of their isoforms (Fig. 2*B*) indicated that common structural properties of the histones contribute to the interaction with the EGCG-modified proteins. Histones can be structurally subdivided into two domains; the globular domain with a rigid three-dimensional structure and the tail domain with a highly charged and conformationally flexible region also called the intrinsically disordered regions (IDRs). To identify a putative binding region in the histone, we prepared maltose-binding protein (MBP)–fused H2B peptides (Fig. 3*A*). An *in vitro* solid-phase binding assay revealed that the N-terminal tail domain of histone H2B (seq. 1–35), but not the C-terminal globular domain (seq. 36–126), should be a binding region of the polyphenol-modified proteins (Fig. 3*B*). The binding activity of the N-terminal region was abolished by acetylation, suggesting that lysine residues abundant in the H2B N-terminal tail domain were involved in the binding to the EGCG-modified proteins. In addition, the EGCG-modified proteins showed the ability to bind to all the shuffled peptides 1, 2, and 3 containing the same amino acids but different sequences (Fig. 3*B*). These results suggested that the EGCG-modified proteins do not bind sequence specifically to the N-terminal region of the histones but to the composition of the positively charged amino acids.

To gain more insight into this hypothesis, we prepared various IDR peptides fused with MBP (Fig. 3*C*) and examined their interaction with the EGCG-modified proteins. The result showed that the EGCG-modified proteins binding was specific for the IDRs containing six or more positively charged amino acids, such as H2B tail, H2A N-tail, H2B F2, H2B F3, H3 tail, H4 tail, and p53 (Fig. 3*D*). Interestingly, only a weak binding was observed for the IDRs, such as H2B F7, which have a large amount of positively charged amino acids but short peptide lengths. In addition, the EGCG-modified proteins did not bind to lysozyme (Fig. S2), which are positively charged but lack the IDRs. These results suggested that not only the positively charged amino acids but also the IDRs are important factors in the binding of the EGCG-modified proteins to histone H2B.

### An oxidized lysine partially contributes to histone binding

**Figure 4:**
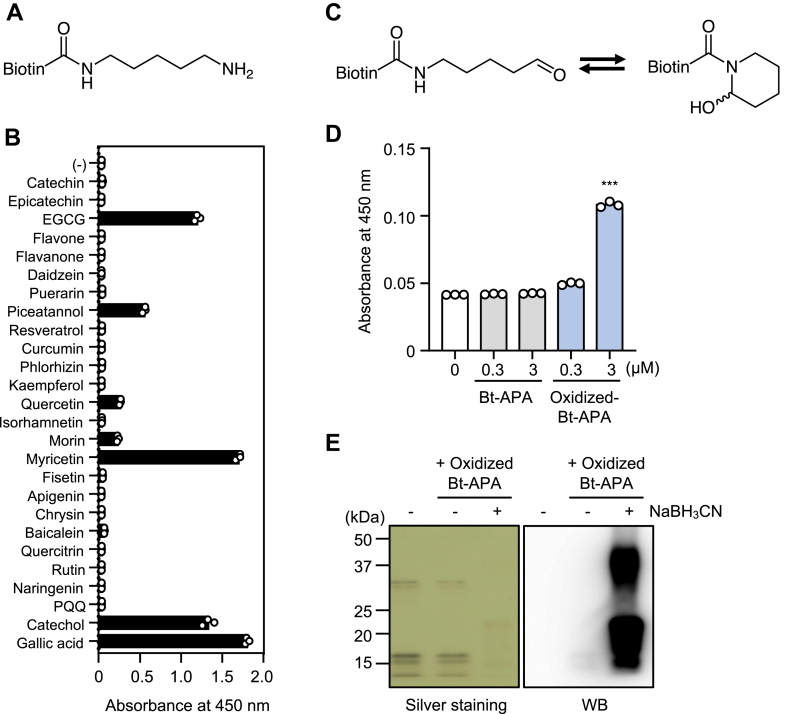
**Oxidized lysine partially contributes to histone binding.***A*, chemical structure of *N*-biotinyl-5-aminopentylamine (Bt-APA). *B*, binding of polyphenol-modified Bt-APA to CTH. CTH immobilized on ELISA plate were incubated with polyphenol-modified Bt-APA (10 μM). The binding was detected using streptavidin-HRP. *C*, chemical structures of the equilibrium products, *N*-biotinyl-5-aminopentanal and *N*-biotinyl-2-piperidinol, provided by oxidative deamination of Bt-APA. *D*, binding of oxidized Bt-APA to CTH. CTH immobilized on ELISA plate were incubated with oxidized Bt-APA. The binding was detected using streptavidin-HRP. Differences were analyzed by Dunnett's test; ∗∗∗*p* < 0.001; *versus* control (0 μM Bt-APA). *E*, Schiff base formation between oxidized Bt-APA and histones was detected by ligand blotting. CTH (100 μg/ml) was incubated with oxidized Bt-APA (300 μM) for 1 h at 37 °C. After incubation, the resulting solutions were reduced with NaBH_3_CN (2 mg/ml) for 24 h at 37 °C. The samples were denatured, separated by SDS-PAGE, transferred to PVDF membranes, and visualized using HRP-conjugated streptavidin. CTH, calf thymus histone; HRP, horseradish peroxidase; PVDF, polyvinylidene difluoride.

EGCG is known to react with lysine residues to form oxidatively deaminated products, such as α-aminoadipic semialdehyde (1). To gain structural insights into the oxidized form of the lysine residues that contribute to the interaction with histone, we used a lysine analog, *N*-biotinyl-5-aminopentylamine (Bt-APA) (Fig. 4*A*), to evaluate the histone-binding activity of the polyphenol-modified lysine analog. As shown in Figure 4*B*, among the 25 plant-derived polyphenols, several polyphenols showed the ability to convert the lysine analog into histone ligands. Based on our recent study on the analysis of the oxidized Bt-APA showing the presence of a unique six-membered ring 2-piperidinol derivative equilibrated with a ring-open product (aldehyde) (Fig. 4*C*) (1, 2, 3), we purified a mixture of aldehyde/2-piperidinol and tested its histone-binding ability. As shown in Figure 4*D*, the oxidized Bt-APA showed a significant histone-binding activity. Thus, the oxidatively deaminated lysine, which is in equilibrium between the ring-open and ring-closed intermediates, may be involved in the interaction of the polyphenol-modified proteins with histones.

On the other hand, based on the identification of the aldehyde/2-piperidinol product as a histone ligand, it was speculated that the binding of the oxidized product to histone might be due to the formation of Schiff base crosslinks between the aldehyde intermediate and histone lysine residues. Hence, CTH incubated with the oxidized Bt-APA was stabilized by reduction with NaBH_3_CN, and the biotin-labeled proteins were detected by Western blotting. Reduction of the mixture with NaBH_3_CN provided a strong signal for biotin, indicating that the oxidized lysine analog might form crosslinks with the histone (Fig. 4*E*). However, no signal was observed during the incubation of EGCG-BSA and H2B, even after reduction with NaBH_3_CN (Fig. S3*A*). We also attempted to detect lysinonorleucine (LNL) as a reduced form of a lysine-derived crosslink, dehydrolysinonorleucine, in the reaction mixture of the EGCG-modified BSA and histone H2B using LC-MS/MS. However, the crosslink was undetectable in the reaction mixture (Fig. S3*B*). These data suggest that oxidatively deaminated products may be involved in the binding to histones through interactions other than the Schiff base crosslinking.

### Involvement of protein-bound polyphenols in histone binding

**Figure 5:**
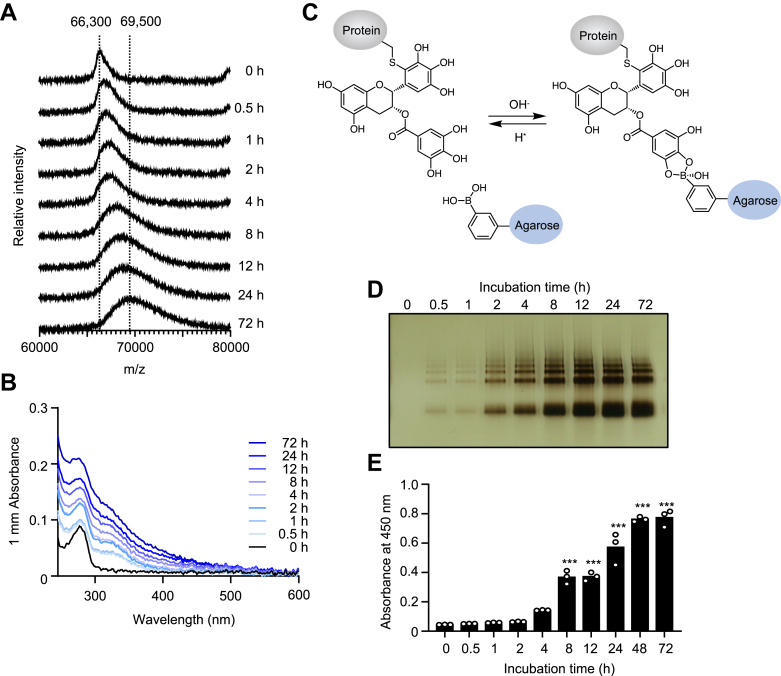
**EGCG forms covalent adducts with proteins.***A*, MALDI-TOF-MS analysis of EGCG-treated BSA. BSA (1 mg/ml) was incubated with 1 mM EGCG and collected each time. The change in the molecular weight was analyzed by MALDI-TOF-MS. *B*, incubation time-dependent changes of the absorbance of BSA incubated with EGCG. 1 mg/ml BSA was incubated with 1 mM EGCG and collected each time, then UV-vis absorbance measured. *C*, schematic representation of PBA purification. EGCG-adducted protein reversibly forms covalent bond with boronate-beads. Lower pH results in releasing EGCG-adducted protein from boronate-beads. *D*, incubation time-dependent binding of EGCG to BSA. 1 mg/ml BSA was incubated with 1 mM EGCG and collected each time. EGCG-modified BSA in the PBA eluate was detected by silver staining. *E*, incubation time-dependent increase of histone-binding ability of BSA incubated with EGCG. 1 mg/ml Bt-BSA was incubated with 1 mM EGCG and collected each time. CTH immobilized on ELISA plate were incubated with EGCG-Bt-BSA (10 μg/ml). The binding was detected using streptavidin-HRP. Differences were analyzed by Dunnett's test; ∗∗∗*p* < 0.001; *versus* 0 h. BSA, bovine serum albumin; CTH, calf thymus histone; EGCG, (-)-epigallocatechin-3-O-gallate; HRP, horseradish peroxidase.

Previous studies have shown that polyphenols form covalent adducts with proteins (1, 4, 5, 6). Hence, we hypothesized that polyphenols bound to proteins might also contribute to the histone binding. To prove this hypothesis, we first measured changes in the molecular weight of EGCG-BSA using MALDI-TOF/MS. As shown in Figure 5*A*, the molecular weight of EGCG-BSA increased over time from about 66,300 to about 69,500, suggesting the addition of multiple molecules of EGCG. The absorbance of EGCG-BSA in the wide range from UV to visible light up to 72 h continuously increased along with the incubation of EGCG and BSA (Fig. 5*B*). This type of spectral broadening is similar to the autoxidation and browning of polyphenols in nature (14). Although it is difficult to define individual chromophores, it can be speculated that polyphenols with different oxidative states may covalently bind to proteins and contribute to the broadening of the absorption over a wide range of wavelengths. A pull-down assay of catechol-containing compounds using boronic acid beads (Fig. 5*C*) showed that an *o*-diphenol structure was time dependently introduced into the protein by EGCG (Fig. 5*D*). Moreover, the introduction of EGCG into the protein correlated with the time-dependent appearance of the histone-binding activity of the EGCG-modified BSA (Fig. 5*E*). These data suggested that protein-bound EGCG may be involved in the binding of the EGCG-modified proteins with histone.

### Involvement of polyphenol polymers bound to proteins in the interaction with histones

**Figure 6:**
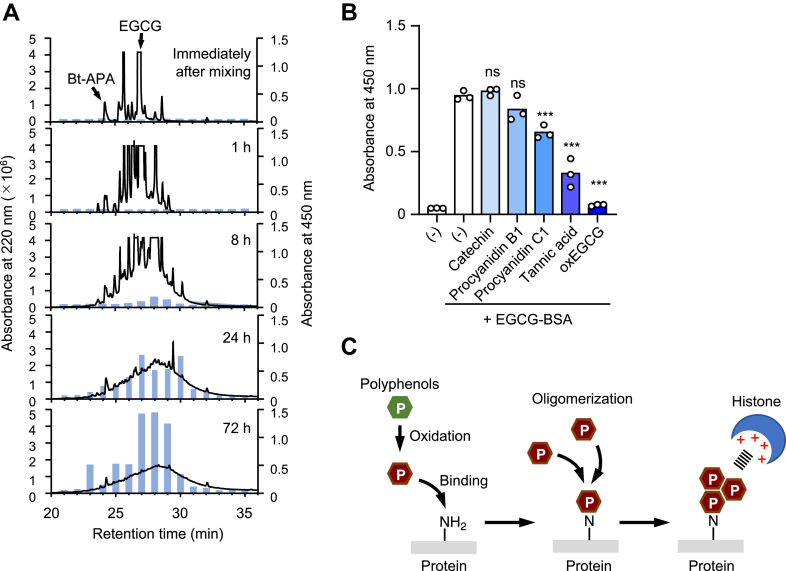
**Adducted multiple EGCG contribute to the histone binding.***A*, time-dependent changes of Bt-APA incubated with EGCG. 1 mM Bt-APA was incubated with 1 mM EGCG at 37 °C. The reaction mixture was separated by HPLC and the absorbance measured at 220 nm (solid line). Each fraction was measured for the binding to CTH by solid-phase binding assay (bar). *B*, competitive inhibition of EGCG-BSA binding to histones by several polyphenols. CTH immobilized on ELISA plate were incubated with EGCG-Bt-BSA (10 μg/ml) in the presence of competitors (50 μM). The binding was detected using streptavidin-HRP. Differences were analyzed by Dunnett's test; ∗∗∗*p* < 0.001; *versus* EGCG-Bt-BSA without competitors. *C*, schematic illustration of the production of histone-binding molecules. Oxidized polyphenols bind to lysine residues in proteins, and protein-bound oligomeric polyphenols might interact with histone. BSA, bovine serum albumin; Bt-APA, N-biotinyl-5-aminopentylamine; CTH, calf thymus histone; EGCG, (-)-epigallocatechin-3-O-gallate; HRP, horseradish peroxidase.

To gain more insight into the protein-bound polyphenols possessing the histone-binding potential, we characterized the EGCG modification of biotin-labeled nucleophilic amino acid analogs and evaluated their histone-binding activity. As shown in Figure 6*A*, EGCG reacted with Bt-APA and immediately gave several unknown products after 1 h of incubation. Further incubation resulted in the appearance of a broad band along with the disappearance of distinct peaks. Of interest, this broad band appeared to show the histone-binding activity. The biotin-labeled cysteine analog, *N*-biotinyl-2-aminoethane-1-thiol (Bt-AET), also reacted with EGCG to produce a heterogeneous compound possessing the histone-binding potential (Fig. S4). These data suggest that a group of complex heterogeneous compounds formed by the reaction of nucleophilic amino acids with EGCG may be involved in the interaction with histone.

On the other hand, EGCG is known to undergo autoxidation and oligomerization in an aqueous solution to form heterogeneous polymers (15). In fact, incubation of EGCG alone gave an HPLC profile similar to the EGCG-treated Bt-APA and Bt-AET (data not shown). Therefore, it was speculated that EGCG polymerized without binding to nucleophilic amino acid analogs might interact with histone. To prove this hypothesis, we tested several polyphenols with various oligomeric states (Fig. S5) as inhibitors for the binding of the polyphenol-modified proteins to histone. As shown in Figure 6*B,* a 3-day-oxidized EGCG (oxEGCG), followed by tannic acid and procyanidin C1, showed a significant inhibitory effect, whereas the monomeric (catechin) and dimeric (procyanidin B1) polyphenols were almost negligible. The inhibitory effect of these polyphenols on the histone-binding activity of polyphenol-modified proteins appeared to correlate with their oligomeric states. Because these oligomeric polyphenols structurally contain several π-electron systems, the protein-bound polyphenols may interact with basic amino acids in the histone tail region through a cation-π interaction (Fig. 6*C*).

### Aggregation of histones with polyphenol-modified proteins

**Figure 7:**
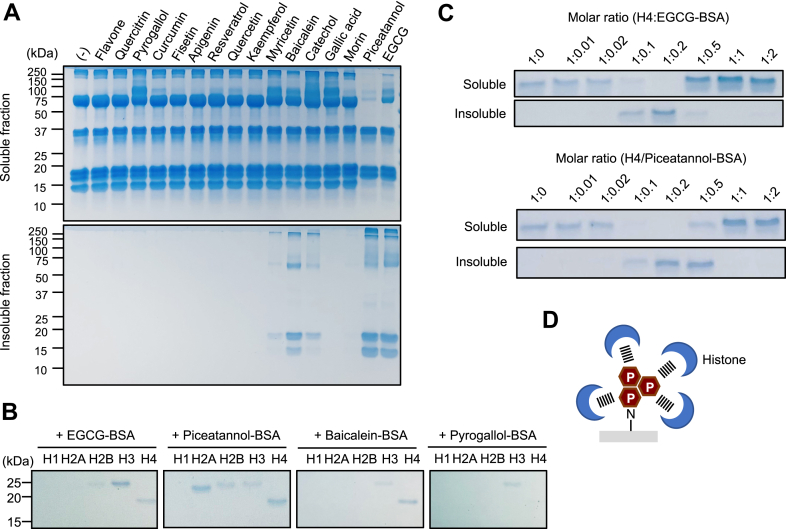
**Polyphenol-modified proteins aggregate with histones.***A*, aggregation of polyphenol-modified proteins with CTH. 1 mg/ml CTH was incubated with 1 mg/ml polyphenol-treated BSA in PBS at room temperature for 10 min. The aggregates were analyzed as described in the Experimental procedures section. *B*, aggregation of polyphenol-modified proteins with recombinant histones. 1 mg/ml recombinant histones (H1, H2A, H2B, H3, and H4) were incubated with 1 mg/ml polyphenol-treated BSA in PBS at room temperature for 10 min. The aggregates were analyzed as described in the Experimental procedures section. *C*, aggregation of polyphenol-modified proteins with recombinant histone H4. 1 mg/ml recombinant histone H4 was incubated with EGCG-BSA (*upper panel*) or piceatannol-BSA (*lower panel*) at various molar ratios in PBS at room temperature for 10 min. The aggregates were analyzed as described in the Experimental procedures section. *D*, schematic illustration of the aggregation of histone with polyphenol-treated proteins. BSA, bovine serum albumin; CTH, calf thymus histone; EGCG, (-)-epigallocatechin-3-O-gallate.

Histones have been shown to form aggregates with serum and plasma proteins, fibrinogen, and Pentraxin 3 (16, 17, 18). Hence, we assessed if histones form aggregates with protein-bound polyphenols. To evaluate the aggregate formation, histones incubated with polyphenol-treated BSA were separated into supernatant (soluble) and precipitate (insoluble) fractions by centrifugation and analyzed by SDS-PAGE. As shown in Figure 7*A*, several polyphenols demonstrated the ability to convert BSA into molecules that form aggregates with CTH. The screening of 16 polyphenols identified 5 polyphenols, including EGCG, piceatannol, and baicalein (Fig. 7*A*), as sources of the aggregates. They are almost the same as those identified as sources of histone ligands (Fig. 2*A*). Histone isoforms, H3 and H4, preferentially formed aggregates with these polyphenol-modified proteins (Fig. 7*B*). To elucidate the mechanisms of aggregation, we investigated the stoichiometry of the formation of the aggregates. Histone H4 was incubated with EGCG-BSA (*upper panel*) or piceatannol-BSA (*lower panel*) at various molar ratios, and after centrifugation, both the soluble and insoluble fractions were analyzed by SDS-PAGE. The aggregates were detectable at the histone/modified proteins molar ratios of 1:0.1 to 0.5 for both EGCG and piceatannol (Fig. 7*C*). In addition, when the molar ratio of the polyphenol-modified proteins was excessive, the histones were recovered from the supernatant. A similar reversible aggregation has been shown to occur in the divalent antibody–antigen complex formation or polyelectrolyte–protein complex formation (19). Therefore, the aggregation of histone and polyphenol-modified proteins may be caused by polyvalent interactions (Fig. 7*D*).

### Inhibition of histone cytotoxicity by polyphenol-modified proteins

**Figure 8:**
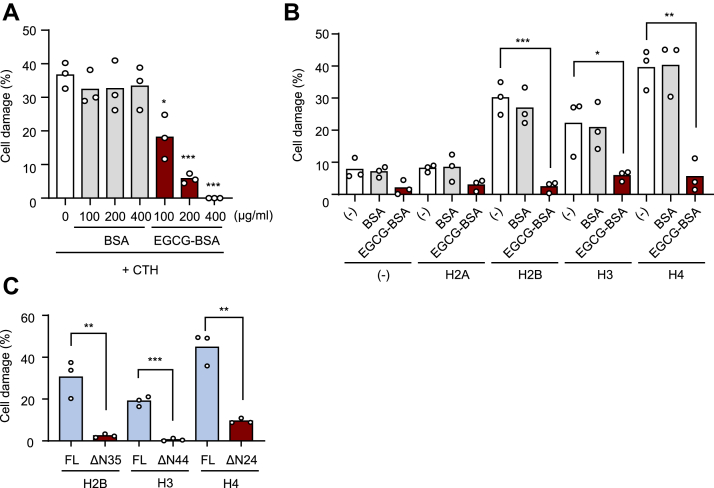
**EGCG-modified proteins mitigate histone-induced cytotoxicity.***A*, effects of EGCG-BSA on CTH-induced cytotoxicity *in vitro*. EA.hy926 cells were incubated with CTH (100 μg/ml) and BSA or EGCG-BSA (100, 200, and 400 μg/ml) for 2 h. The cell damage rate was measured by an LDH assay. Differences were analyzed by Dunnett's test; ∗*p* < 0.05; ∗∗∗*p* < 0.001; *versus* control (0 μg/ml). *B*, effects of EGCG-BSA on recombinant histone–induced cytotoxicity *in vitro*. EA.hy926 cells were incubated with recombinant histones (H2A, H2B, H3, and H4) (100 μg/ml) and BSA or EGCG-BSA (100, 200, and 400 μg/ml) for 2 h. The cell damage rate was measured by an LDH assay. Differences were analyzed by Dunnett's test; ∗*p* < 0.05; ∗∗*p* < 0.01; ∗∗∗*p* < 0.001; *versus* control (−). *C*, contribution of histone tail region to histone-induced cytotoxicity. EA.hy926 cells were incubated with recombinant full-length histones (H2B, H3, and H4) or tail-deficient histones (H2B-ΔN35, H3-ΔN44, and H4-ΔN24) (100 μg/ml) for 2 h. The cell damage rate was measured by an LDH assay. Differences were analyzed by Dunnett's test; ∗∗*p* < 0.01; ∗∗∗*p* < 0.001; *versus* FL. BSA, bovine serum albumin; CTH, calf thymus histone; EGCG, (-)-epigallocatechin-3-O-gallate; LDH, lactate dehydrogenase.

Extracellular histones penetrate cell membranes and exhibit cytotoxicity due to positively charged amino acid residues in the N-terminal tail region (12). Such histone cytotoxicity results in exacerbation of atherosclerosis through plaque destabilization, whereas neutralizing agents for the histones blocked histone penetration to reduce the cytotoxicity. Because polyphenol-modified proteins also bind to the histone tail, we hypothesized that they might reduce the histone cytotoxicity through binding to the histones. CTH showed a cytotoxicity to the vascular endothelial cell line EA.hy926, which was attenuated by polyphenol-modified proteins in a concentration-dependent manner (Fig. 8*A*). Not only the EGCG-modified proteins but also other polyphenol-modified proteins showing the histone-binding ability reduced the histone cytotoxicity (Fig. S6). The protein-bound polyphenols showed a significant inhibitory effect on the cytotoxicity induced by H2B, H3, and H4 (Fig. 8*B*). Assessing the cytotoxicity of the tail-deficient mutants of H2B, H3, and H4, loss of the tail region significantly reduced the cell damage (Fig. 8*C*). These findings suggest that the polyphenol-modified proteins can reduce histone-induced cytotoxicity through binding to the histone tail region.

## Discussion

Antioxidants are sensitive to oxidation and are quickly converted to an oxidized form after exhibiting an antioxidant function. An interesting part of this reaction is that oxidized antioxidants react with biological molecules such as proteins. In addition, proteins modified with antioxidants gain a new function. Antioxidants, such as vitamin C and polyphenols, convert proteins into innate epitopes that can be recognized by natural IgM antibodies (1, 2, 3, 20). The oxVC-modified proteins have been shown to bind to histone localized on the cell surface (13). These findings led to the prediction that antioxidants may exert a similar activity through conversion to their oxidized forms. Indeed, this study showed that several polyphenols could convert proteins to histone ligands. Further mechanistic studies showed the involvement of a complex heterogeneous group of compounds bound to proteins as histone-binding elements. In addition, we characterized the formation of aggregates resulting from a polyvalent interaction between the polyphenol-modified proteins and histones and investigated the protective effect of polyphenol-modified proteins on the endothelial cytotoxicity of histone. These findings established that histones are one of the major targets of polyphenol-modified proteins and provide important insights into the chemopreventive functions of dietary polyphenols.

In the present study, we first screened 25 polyphenols for the conversion of BSA to histone ligands and identified several polyphenols, including EGCG, piceatannol, and baicalein (Fig. 2). The data showed that compounds with pyrogallol moieties, such as baicalein, EGCG, gallic acid, and myricetin, consistently exhibit histone-binding activity after incubation with BSA. Therefore, pyrogallol is likely the key structure required for these transformations. Pyrogallol-containing polyphenols have been reported to readily auto-oxidize to produce unstable species with *o*-quinone structures (21). It has been proposed that these pyrogallol-derived species may react directly with primary amino groups of lysine residues (1). On the other hand, some compounds with catechol moieties, such as catechol, morin, and piceatannol, also exhibited an activity, while other catechol compounds did not. Although the reason for these different activities among catechol-containing polyphenols remains unclear, we predict that it may be due to how well they can be converted to pyrogallol-containing species during incubation.

Based on the results that the EGCG-modified proteins are bound to histone proteins regardless of their isoforms, it was speculated that the common structural properties of histones contribute to the interaction with the EGCG-modified proteins. In this regard, within the two domains (globular and N-terminal tail domains), we identified the N-terminal tail domain of histone H2B as a binding region for the polyphenol-modified proteins (Fig. 3). This domain contains the positively charged, conformationally flexible region called IDR. Solid-phase binding studies using MBP-fused IDR peptides revealed that EGCG-BSA binds to several IDRs containing large numbers of positively charged amino acid residues. In addition, the result that the EGCG-modified proteins showed the ability to bind to all the shuffled peptides containing different sequences with the same amino acids suggest that the number of positively charged amino acids may be important for the interaction of the modified proteins with the N-terminal region of the histones. Therefore, it was concluded that an important element in histone proteins must be the N-terminal IDR. IDRs are involved in a variety of biological responses, including mediating biomolecular assembly, providing ligand binding sites, and supporting protein folding by acting as chaperones (22). Thus, our findings suggested that polyphenol-modified proteins may exert a wide range of biological functions by binding not only to histones but also to various cationic IDRs.

Catechol-type polyphenols oxidatively deaminate lysine to produce an equilibrium product of an aldehyde and 2-piperidinol (2). Aldehyde/2-piperidinol derivatives showed a significant histone-binding activity (Fig. 4), suggesting that the initial oxidation products of lysine may be partially involved in the interaction of the polyphenol-modified proteins with the histones. The aldehyde derivative equilibrated with the 2-piperidinol product was also expected to react with an α-amino group of an unoxidized lysine residue in histones to form a lysine-derived crosslink *via* a Schiff base (3). However, the crosslink was not detected in the EGCG-modified BSA incubated with the histones (Fig. S3). Therefore, the contribution of the Schiff base crosslinking in the interaction between the aldehyde intermediates and histone lysine residues is unknown. The 2-piperidinol derivative equilibrated with the aldehyde product may interact noncovalently with the histones. However, the detailed mechanism of the involvement of the oxidatively deaminated lysine residues in the interaction of the EGCG-modified proteins with the histones remains unclear.

Polyphenol modification of proteins is also known to be associated with astringency, which is thought to be caused by the interaction of polyphenols with basic salivary proline-rich proteins. Jöbstl *et al*. (23) proposed a molecular model for astringency produced by polyphenol/protein interactions as follows: (i) small soluble polydispersed particles are formed at low EGCG ratios, which aggregate to form larger particles as EGCG is added. (ii) There is an initial compaction of the protein as it binds to the polyphenol. (iii) The particle subsequently increases in size as EGCG is added because of the incorporation of EGCG, then to aggregation and precipitation. Based on these previous studies, we investigated the involvement of the protein-bound polyphenols in the binding to histone. A pull-down assay using boronic acid beads indeed showed a time-dependent introduction of EGCG into the protein, which correlated with the appearance of the histone-binding activity of the EGCG-modified protein (Fig. 5). In addition, we characterized the EGCG modification of the lysine and cysteine analogs and found that a group of complex heterogeneous compounds might contribute to the interaction with histone. These heterogenous derivatives are known to be formed by a mechanism similar to the progressive self-assembly of catecholamines (24). Indeed, several polyphenols with different oligomeric states showed significant inhibitory effects on the binding of the EGCG-modified proteins to the histones (Fig. 6). Thus, the actual molecules contributing to the interaction with histone may be the polyphenol oligomers formed on the protein.

The covalent binding of polyphenols to proteins also represents the introduction of a phenol-derived π-electron system into proteins. The introduction of the π-electron system allows proteins to interact with basic amino acids, such as lysine and arginine, through cation-π interactions (25). Therefore, it can be speculated that the covalent binding of polyphenols introduces a π-electron system to the protein, leading to cation-π interactions with basic amino acids in histones. This study revealed that high affinity interactions between histone and polyphenol-modified proteins form aggregates (Fig. 7). The stoichiometry of aggregate formation demonstrated the soluble–insoluble transition of histone H4 by EGCG-BSA. Histone/EGCG-BSA precipitation occurred at low concentrations of EGCG-BSA and the histone/EGCG-BSA complex was solubilized by the increasing EGCG-BSA concentrations. Aggregation of histone by EGCG-BSA may be explained as follows: at low EGCG-BSA concentrations, the interaction between histone and EGCG-BSA is favorable due to charge neutralization of the proteins and EGCG-BSA, resulting in the formation of histone/EGCG-BSA aggregates. At high concentrations of EGCG-BSA, an excess amount of EGCG-BSA decreases the attraction between histone and EGCG-BSA due to a decline in the number of protein molecules per unit of EGCG-BSA. Thus, proteins modified by certain polyphenols may act as polyelectrolytes. This hypothesis is supported by the fact that EGCG treatment results in the increased negative charge of the proteins (1).

Ullmann *et al*. (26) have shown that a single dose administration of 500 to 1600 mg EGCG in mice results in a mean maximum plasma concentration of 6.4 μM in 2 h with no clinical or biological adverse events. Therefore, EGCG used in this study is in excess over protein. However, in our preliminary experiments (Yamaguchi, K., Itakura, M., and Uchida, K., unpublished observations), binding of Bt-EGCG to serum proteins was observed when mouse sera were incubated with 10 μM Bt-EGCG for 3 h. In addition, significant binding of Bt-EGCG–modified proteins to histones was also observed. These data suggest that the micromolar concentration of polyphenols may be sufficient to modify plasma proteins and induce histone binding *in vivo*.

Extracellular histones released from leucocytes have been implicated in pathogenesis, such as inflammation (27). Histones interact with the negative charges of the cell membrane through their positively charged tail regions, causing the cell membrane to collapse (12). We found that EGCG-BSA protects endothelial cells from extracellular histone-mediated cytotoxicity *in vitro* (Fig. 8). A mechanism underlying the inhibition of histone cytotoxicity was suggested to be due to the interaction of EGCG-BSA with the tail region of the histones. Thus, this study adds polyphenol-modified proteins as potential inhibitors of extracellular histone-mediated cell death. Moreover, the cytoprotective effects of the polyphenol-modified proteins could explain the chemopreventive functions of polyphenols for chronic diseases. In atherosclerosis, histone H4 released from neutrophils lyses smooth muscle cells and destabilizes atherosclerotic plaques. However, histone inhibitory peptides, containing a large amount of negatively charged amino acids and forming complexes with histone H4 tails, have been shown to inhibit the histone-induced cytotoxicity and increase the stability of the plaques (12). Polyphenol-modified proteins, which can interact with the histone tail lesion as well as histone inhibitory peptide, may also contribute to mitigate chronic inflammation.

In conclusion, this study identified polyphenol-modified proteins as a potential histone ligand. Mechanistic studies have shown the involvement of complex heterogeneous groups of polyphenol-derived compounds bound to proteins as histone-binding elements. The interaction of polyphenol-modified proteins with histones has been shown to form aggregates and provide a protective effect against the histone-mediated cytotoxicity of endothelial cells. The results of this study suggest that many of the beneficial effects of polyphenols may be due, at least in part, to the formation of protein-bound polyphenols and their interaction with histones. However, despite the association between the polyphenol modification of proteins and astringency, the presence of protein-bound polyphenols has not been established. Therefore, future studies need to evaluate the formation of protein-bound polyphenols *in vivo* and investigate the contribution of this unique molecule to human health and diseases.

## Experimental procedures

### Modification of proteins and biotin-labeled amino acid analogs

Biotin-labeled proteins were prepared by incubating 5 mg/ml BSA (Iwai Chemicals), human serum albumin (FUJIFILM Wako Pure Chemical, Inc), transferrin (FUJIFILM Wako Pure Chemical, Inc), and IgG (FUJIFILM Wako Pure Chemical, Inc) with a 10-fold molar Bt-PE-maleimide (Dojindo Laboratories) in PBS (10 mM sodium phosphate buffer (pH 7.4)) at 25 °C for 16 h. After incubation, the aliquots were dialyzed against PBS. The polyphenol-modified proteins were prepared by incubating 1 mg/ml proteins or biotinylated proteins with 2.5 mM polyphenols in PBS containing 10% dimethyl sulfoxide for 72 h at 37 °C under atmospheric oxygen. After incubation, the reactants were collected and dialyzed with PBS. The protein concentrations were measured by a bicinchoninic acid assay (Nacalai Tesque, Inc). Bt-AET was synthesized according to previous studies (28). The polyphenol-modified amino acid analogs were prepared upon incubation of 1 mM Bt-APA (Thermo Fisher Scientific, Inc) or Bt-AET with 2.5 mM polyphenols in PBS containing 10% dimethyl sulfoxide for 24 h at 37 °C under atmospheric oxygen. The oxidized Bt-APA was prepared according to a previous report (2).

### Expression and purification of recombinant histones and MBP-fusion peptides

The pET15b vectors carrying the His-tagged human histones H2A, H2B, H3, and H4 and the pET21a vector carrying the His-tagged human histone H1.2 were kindly provided by Dr Kurumizaka (The University of Tokyo, Japan). The expression and purification of the recombinant histones were performed according to previous reports (29, 30, 31). Briefly, the *Escherichia coli* BL21 (DE3) cells were freshly transformed with the vectors described previously and grown on LB plates containing ampicillin (100 μg/ml) at 37 °C. After a 16 h incubation, the colonies were inoculated into LB medium containing ampicillin. After overnight cultivation, the cells were harvested and resuspended in 50 mM Tris–HCl (pH 8.0), 500 mM NaCl, 1 mM PMSF, and 5% glycerol. The cells were disrupted by two rounds of sonication for 15 min each. The cell lysates were centrifuged at 18,000*g* for 20 min at 4 °C. The His-tagged histones H2A, H2B, H3, and H4 were recovered in the insoluble pellets, while H1 was in the supernatant. The pellets were dissolved in 50 mM Tris–HCl (pH 8.0), 500 mM NaCl, 1 mM PMSF, 5% glycerol, and 7 M guanidinium chloride by sonication and overnight incubation at 4 °C. After centrifugation, the supernatants containing the His-tagged histones were mixed with Ni-NTA agarose beads (FUJIFILM Wako Pure Chemical, Inc), and the samples were rotated at 4 °C for 60 min. The beads were packed into Econo-columns (Bio-Rad) and washed with 50 mM Tris–HCl (pH 8.0), 500 mM NaCl, 1 mM PMSF, 5% glycerol, 6 M urea, and 5 mM imidazole. The His-tagged histones were eluted with the same buffer except that the concentration of imidazole was 500 mM. The eluates were dialyzed overnight against 5 mM Tris–HCl (pH 8.0) and 2 mM 2-mercaptoethanol at 4 °C.

For purification of the MBP-fusion peptides, the peptide sequences were amplified and cloned into the pMAL-p2X expression vector. *E. coli* BL21 (DE3) cells were transformed with vectors and isolated colonies cultured in LB medium containing ampicillin (100 μg/ml) at 37 °C. After overnight cultivation, the cells were inoculated in 200 ml of LB medium and incubated at 37 °C until the *A*_600_ reached 0.4 to 0.5 when the expression was induced by the addition of IPTG to a final concentration of 0.3 mM. After a 4 h incubation, the cells were centrifuged and the bacterial pellets were resuspended with an osmotic shock solution (30 mM Tris–HCl, 20% sucrose, 1 mM EDTA, pH 8.0). The suspensions were incubated for 10 min with shaking followed by centrifugation at 8000*g* for 20 min at 4 °C. The supernatants were removed, the pellets were resuspended in ice-cold 5 mM MgSO_4_, incubated for 10 min on ice, and centrifuged at 8000*g* at 4 °C for 20 min. The supernatants were supplemented with 1 M Tris–HCl, pH 7.4, to a final concentration of 20 mM, applied to amylose resin columns, washed with column buffer (20 mM Tris–HCl, 200 mM NaCl, 1 mM EDTA, pH 7.4), and eluted with elution buffer (20 mM Tris–HCl, 200 mM NaCl, 1 mM EDTA, 10 mM maltose, pH 7.4). The fractions containing the MBP fusion-peptides were pooled and dialyzed against PBS.

### Solid-phase binding assay

Histone from calf thymus (Thermo Scientific) (10 μg/ml), the recombinant histones (10 μg/ml), MBP-fused histones (1 μM), and lysozyme (Thermo Scientific) (10 μg/ml) were immobilized overnight onto 96-well MaxiSorp plates (Nunc) at 4 °C. The plates were blocked with 2% skim milk in PBS and Tween 20 (PBST) for 1 h, washed three times with PBST, and then either of the biotin-labeled compounds were added to the wells. After a 1 h incubation, streptavidin-horseradish peroxidase (HRP) in PBST was added, then incubated for 1 h. For the solid-phase binding assay using the MBP-fused peptides, biotin-labeled compounds and streptavidin-HRP were preincubated for 5 min. HRP was then developed using the TMB Ultra substrate (Thermo Scientific). The binding of the biotin-labeled compounds to histones was quantified by measuring the absorbance at 450 nm. For the competitive assay, EGCG-Bt-BSA was incubated with 50 μM polyphenols for 1 h on CTH-coated plates.

### Acetylation of MBP-fused H2B N-terminal tails

Acetylation of the MBP-fused H2B N-terminal tails was performed according to previous reports (32). Briefly, 100 μl of a saturated solution of sodium acetate was added to 100 μl of 1 mg/ml MBP-fused H2B N-terminal tails on ice. Two microliters of acetic anhydride was then added to the sample solution and stirred for 30 min on ice. Finally, the sample solution was dialyzed against PBS.

### Ligand blotting

CTH (100 μg/ml) or H2B (100 μg/ml) was incubated with oxidized Bt-APA (300 μM) or EGCG-Bt-BSA (100 μg/ml) for 1 h at 37 °C. After incubation, the resulting solution was reduced with NaBH_3_CN (2 mg/ml) for 24 h at 37 °C. The protein samples underwent SDS-PAGE. After electrophoresis, the gel was transblotted onto a polyvinylidene difluoride membrane (GE Healthcare), incubated with a blocking one (Nacalai Tesque, Inc), washed, and then incubated with streptavidin-HRP (1:5000) for 1 h at room temperature (RT). This procedure was followed by the addition of the SuperSignal West Pico PLUS Chemiluminescent Substrate (Thermo Fisher Scientific, Inc). The blots were visualized by ImageQuant LAS4000 (Cytiva).

### Detection of LNL by LC-electrospray ionization-MS/MS

H2B (100 μg/ml) was incubated with oxidized EGCG-BSA (100 μg/ml) for 1 h at 37 °C. After incubation, the resulting solution was reduced with NaBH_3_CN (2 mg/ml) for 24 h at 37 °C. The protein samples were then hydrolyzed with 6 N HCl for 24 h at 110 °C. The acid-hydrolyzed samples were subjected to an LC-electrospray ionization (ESI)-MS/MS analysis using the TQD-ACQUITY UPLC system (Waters). The sample injection volumes of 5 μl each were separated on an Intrada amino acid column (100 mm × 2.0 mm) (Imtakt) at the flow rate of 0.3 ml/min. A discontinuous gradient of solvent A (acetonitrile containing 0.1% formic acid) with solvent B (100 mM ammonium formate) was used as follows: 10% B at 0 min, 10% B at 2 min, 100% B at 8 min, and 100% B at 10 min. Multiple reaction monitoring was performed in the positive ion mode using nitrogen as the nebulizing gas. The experimental conditions were as follows: ion source temperature, 120 °C; desolvation temperature, 350 °C; cone voltage, 30 eV; collision energy, 20 eV; desolvation gas flow rate, 800 l/h; cone gas flow rate, 50 l/h; collision gas, argon. The strategy was designed to detect the product ion (m/z 84.0) from the positively ionized LNL (m/z 276.0). We synthesized the LNL standard according to a previous report (3).

### PBA pull down of EGCG-BSA

We isolated the EGCG-bound proteins by a specific interaction between catechols and boronic acid using PBA according to previous reports with a minor change (4, 33). A sample of 100 μg total protein was mixed with 100 μl of PBA in 1 ml of 100 mM Tris (pH 8.6) containing 0.1% Triton X-100. The mixture was incubated overnight at 4 °C with rotary shaking. The resin was then washed with 2 ml of 1 mM Tris–HCl (pH 8.6), 4 ml of 250 mM Tris (pH 6.5), and 2 ml of 1 mM Tris (pH 6.5). The proteins were released from the boronate resin by elution with 1 ml of 50 mM glycine (pH 2.0). The protein samples then underwent nondenaturing PAGE (native-PAGE).

### MS analysis of EGCG-BSA

A MS analysis of the unmodified BSA and EGCG-BSA was performed using a MALDI-TOF/TOF mass spectrometer (Autoflex Speed MALDI TOF/TOF system, Bruker Daltonics) operated in the positive ion linear mode.

### UV-visible absorption analyses

The UV-visible absorbance of EGCG-BSA was measured using a Nanodrop 2000.

### HPLC

The EGCG-treated biotin-labeled amino acid analogs were prepared upon incubation of Bt-APA or Bt-AET (1 mM) with EGCG (1 mM) at 37 °C for 24 h. For the fractionation using HPLC, the reaction mixture of EGCG-Bt-APA or EGCG-Bt-AET (500 μl) was analyzed by reverse-phase HPLC on a ChromaNik Sunniest C-18 column (6.0 mm × 250 mm, ChromaNik) at the flow rate of 1.0 ml/min. A gradient of solvent A (water containing 0.1% TFA) with solvent B (acetonitrile containing 0.1% TFA) was used as follows: 5% B at 0 to 10 min, 95% B at 40 min, and 95% B at 45 min. The elution profiles were monitored by absorbance at 220 nm. Samples were separated into 15 fractions from 20 to 35 min (1 min/fraction). Each fraction was lyophilized and reconstituted in 300 μl PBS and subjected to a solid-phase binding assay.

### LC-ESI-MS

Each of the separated fractions by HPLC was subjected to an LC-ESI-MS analysis using a SQD single quadrupole mass spectrometer (Waters) equipped with the ACQUITY ultraperformance LC (UPLC) system (Waters). The sample injection volumes of 5 μl each were separated on Develosil HB C30-UG (2.0 mm × 100 mm, Nomura) at the flow rate of 0.3 ml/min. A gradient of solvent A (water) with solvent B (acetonitrile) was used as follows: 5% B at 0 min, 95% B at 8 min, and 95% B at 13 min. The MS scan was performed in the positive ion mode using nitrogen as the nebulizing gas. The experimental conditions were as follows: ion source temperature, 120 °C; desolvation temperature, 350 °C; cone voltage, 20 eV; desolvation gas flow rate, 800 l/h.

### Synthesis of biotin-labeled EGCG

The biotin-labeled EGCG (Bt-EGCG) was prepared upon incubation of Bt-AET (1 mM) with EGCG (1 mM) at 37 °C for 1 h. For the fractionation using HPLC, the reaction mixture of EGCG-Bt-AET (500 μl) was analyzed by reverse-phase HPLC on a ChromaNik Sunniest C-18 column (10.0 mm × 250 mm, ChromaNik) at the flow rate of 4.7 ml/min. A gradient of solvent A (water containing 0.1% TFA) with solvent B (acetonitrile containing 0.1% TFA) was used as follows: 5% B at 0 to 10 min, 51% B at 25 min, 95% B at 30 min, and 95% B at 35 min. The elution profiles were monitored by absorbance at 220 nm. Fractions corresponding to the peak of Bt-EGCG were dried and stored at -80 °C. Bt-EGCG was characterized by ESI-MS and ^1^H NMR. Bt-EGCG: ESI-MS 761 (M + H)^+^; ^1^H NMR (500 MHz, Methanol-d4): δ1.32 to 1.54 (m, 6H), 1.91 to 2.10 (m, 2H), 2.64 (m, 1H), 2.84 to 3.21 (m, 6H), 4.22 (m, 1H), 4.42 (m, 1H), 5.64 (m, 2H), 5.95 (m, 1H), 6.72 (s, 1H), and 6.89 (m, 2H). 3.28 (2H) and 4.84 (1H) were masked by solvent peaks.

### Mice

All experiments were performed according to the guidelines of the Animal Usage Committee of the Faculty of Agriculture, The University of Tokyo, and were approved by the committee (Permission No. P19-047). Male BALB/c mice were purchased from Japan SLC. The mice were housed in a temperature-controlled pathogen-free room with light from 8:00 to 20:00 (daytime) and had free access to standard food and water. Blood was collected from the tail vein and allowed to stand for 1 h at RT, after which the sera were collected by centrifugation at 1000*g* for 10 min and stored at −80 °C until used. The Bt-EGCG-treated mouse sera were prepared by incubating the sera with 10 μM Bt-EGCG at 37 °C under atmospheric oxygen.

### Histone aggregation assay

One milligram per milliliter CTH or recombinant histones (H1, H2A, H2B, H3, and H4) was incubated with 1 mg/ml polyphenol-treated BSA in PBS at RT for 10 min. The incubation mixtures were then centrifuged at 12,000*g* for 10 min, and the resultant supernatants and pellets were collected as the “Soluble fraction” and “Insoluble fraction,” respectively. The proteins in each fraction were separated by SDS-PAGE and stained with Coomassie brilliant blue.

### Cytotoxicity assay

To determine the effects of the polyphenol-modified BSA on the histone-induced cytotoxicity in human endothelial EA.hy926 cells, the cell damage rates were evaluated using the lactate dehydrogenase assay Kit (FUJIFILM Wako Pure Chemical, Inc). Briefly, the cells were seeded on a 96-well plate and grown for 24 h. The cells were incubated with 100 μg/ml histone plus 400 μg/ml polyphenol-modified BSA. The cells were incubated at 37 °C for 2 h and the released lactate dehydrogenase EA.hy926 was quantified according to the manufacturer’s instructions.

## Data availability

All data are included within this article.

## Supporting information

This article contains the supporting information.

## Conflict of interest

The authors declare that they have no conflicts of interest with the contents of this article.
