# Supplementary material for: Natural polyphenols convert proteins into histone-binding ligands
Source: J Biol Chem. 2022 Sep 24;298(11):102529. doi: 10.1016/j.jbc.2022.102529 (PMC9589214; doi:10.1016/j.jbc.2022.102529)

Supplementary Figure 1

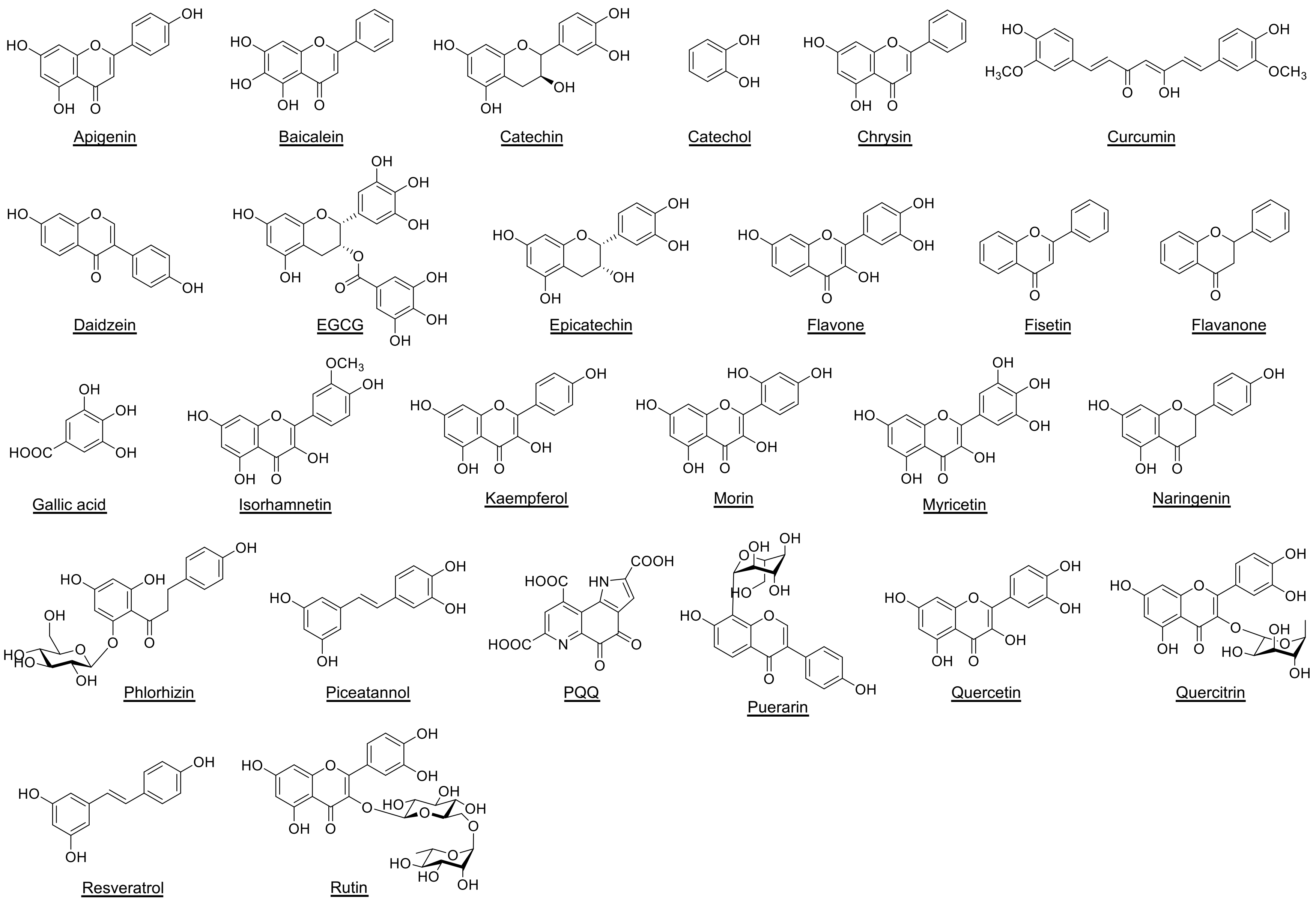

Supplementary Figure 2

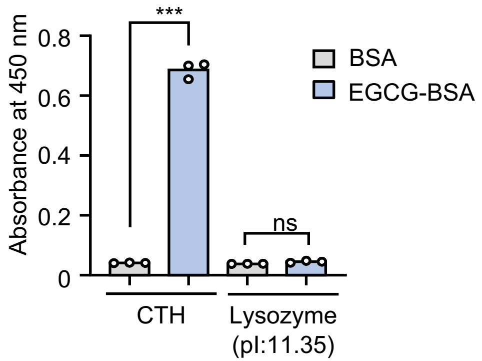

Supplementary Figure 3

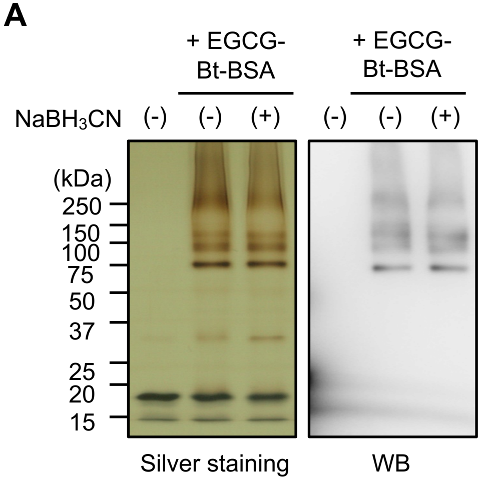

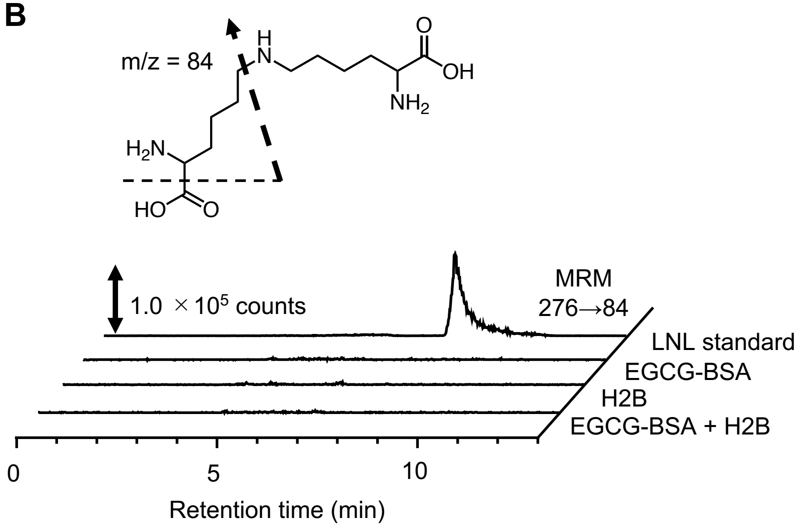

Supplementary Figure 4

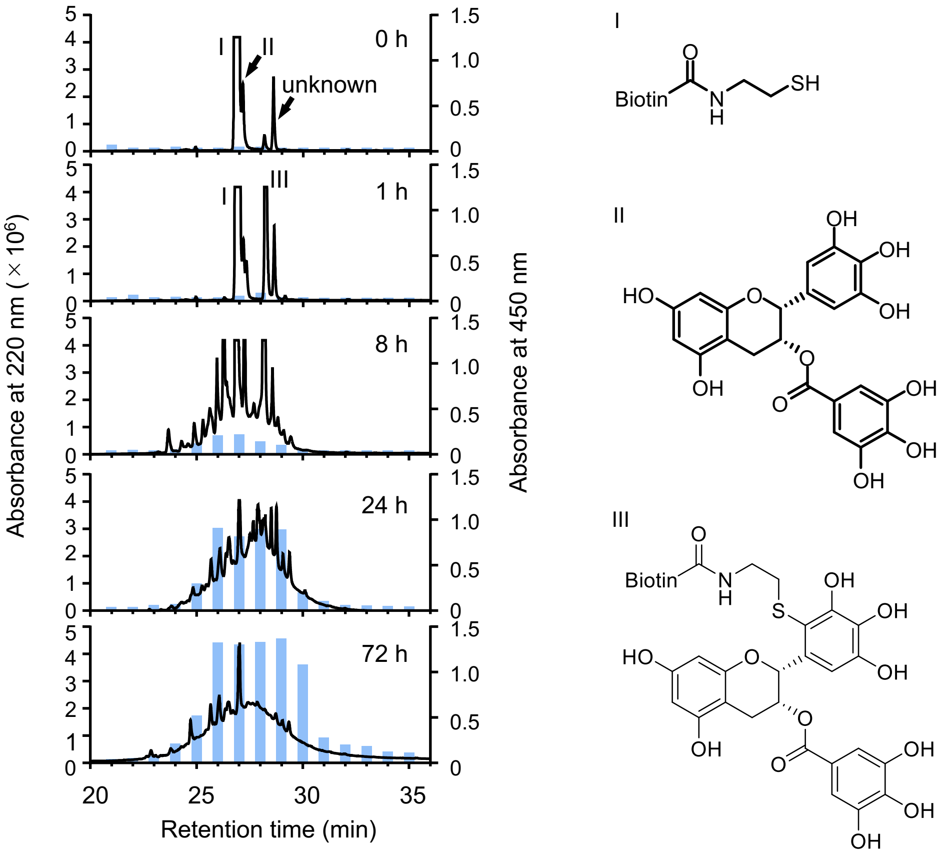

Supplementary Figure 5

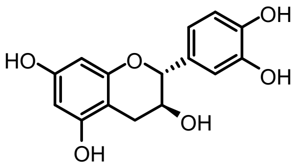

Catechin

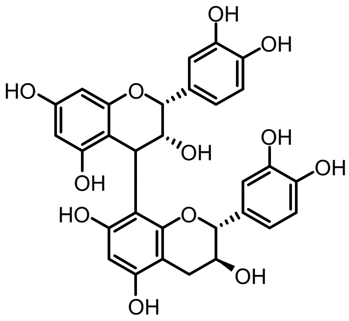

Procyanidin B1

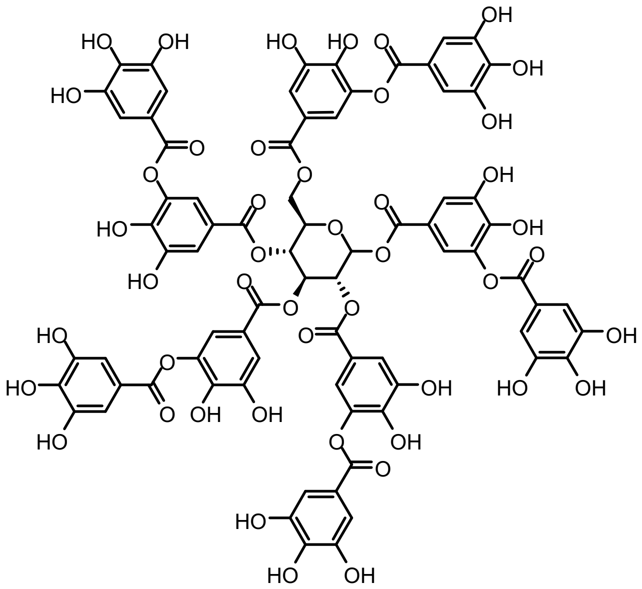

Tannic acid

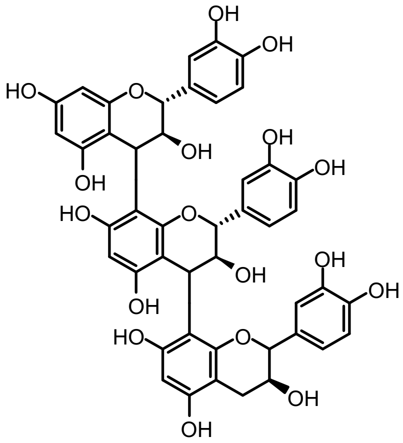

Procyanidin C1

Supplementary Figure 6

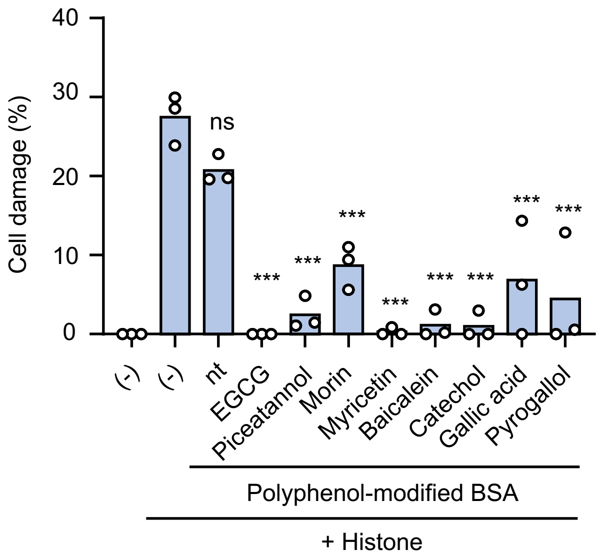

Supplement: Supplemental data [file mmc1.pdf]
